# Editorial: Characterization, Biosynthesis and Biological Functions of Novel Glyco-Epitopes

**DOI:** 10.3389/fmolb.2022.871037

**Published:** 2022-03-04

**Authors:** Shi Yan, Adnan Hodžić, Giulia Bandini, Ganglong Yang, Jorick Vanbeselaere, Yann Guérardel

**Affiliations:** ^1^ Institute of Parasitology, University of Veterinary Medicine Vienna, Vienna, VIA, Austria; ^2^ York Biomedical Research Institute and Department of Biology, University of York, York, United Kingdom; ^3^ Key Laboratory of Carbohydrate Chemistry and Biotechnology, Jiangnan University, Wuxi, China; ^4^ Department of Chemistry, University of Natural Resources and Life Sciences, Vienna, VIA, Austria; ^5^ Université de Lille, CNRS, UMR 8576 - UGSF - Unité de Glycobiologie Structurale et Fonctionnelle, Lille, France; ^6^ Institute for Glyco-core Research (iGCORE), Gifu University, Gifu, Japan

**Keywords:** glycosylation, glycomics, glyco-epitope, mass spectrometry, α-gal syndrome

Complex and diverse glycans are ubiquitous to all cells and essential to all life forms (Varki, 2017). In contrast to nucleic acids, proteins and lipids, glycans represent perhaps the most structurally diverse macromolecules of life. In both prokaryotic and eukaryotic cells, monosaccharides are enzymatically converted to lipid and nucleotide sugar donors as building blocks, which are transported in the latter to different cell compartments by specific nucleotide sugar transporters, and assembled by glycosyltransferases into large glycan molecules (oligo-/polysaccharides) that play important biological roles either in their free forms or by attaching to proteins and lipids to form glycoconjugates.

Glycans can be recognized as ligands by glycan-binding proteins (GBPs), which include anti-glycan antibodies, different classes of lectins, membrane-bound cell receptors, and toxins. A GBP often binds to either one or a few sugar residues of an intact glycan. For example, the influenza hemagglutinin binds to either α2,3- or α2,6-linked sialic acids on the cell surface, whereas some selectins target the tetrasaccharide sialyl-Lewis X epitope (Olofsson and Bergström 2005; Varki et al., 2015). Therefore, it is important to define the precise structures of glyco-epitopes and to understand their biosynthetic basis. Analytical tools, particularly high-resolution mass spectrometry, have rapidly developed in the past decades and have allowed structural characterization of glycans and glycoconjugates in detail.

The current knowledge on glyco-epitopes and their receptors are primarily about mammalian species. Human glycomes have been extensively studied and keep being subject of research that aims at characterizing the changes in glycans associated with different diseases. On the other hand, the glycomes of many other organisms are less understood. Many non-mammalian species have rather different glycosylation machinery, capable of synthesizing unusual glycan structures. Furthermore, the unique structures made by pathogens can be immunogenic to mammals and humans, *e.g.,* by acting as ligands for cell receptors involved in innate immunity or inducing specific anti-glycan antibodies.

Below, we introduce the six manuscripts within this series, which cover a broad range of glycans, and discuss the analytical, biochemical and bioinformatic analysis of different epitopes as well as the medical application potential of the α-Gal epitope.

## Advanced Analytical Approaches Enable Identification of Novel Glyco-Epitopes and Add Rich Details to the Glycomes

Alterations of protein glycosylation during disease progression have been described in many previous studies, particularly in cancer and autoimmune diseases. Consequently, glycans represent novel pathological biomarkers for the early diagnosis of diseases (Dube and Bertozzi 2005). Seo et al. investigated *N*-glycan profiles of sera samples taken from healthy donors and patients diagnosed with Behçet’s disease (BD), a rare immune disorder that can cause inflammation in multiple parts of the body. Native *N*-glycans of serum glycoproteins were released and analyzed by MALDI-TOF MS for glycoform profiling and PGC-LC-MS for isomer-specific monitoring of sialylated glycans. Based on their sensitivity to α2,3-sialidase and elution patterns on PGC-LC using reference glycans carrying α2,6-sialic acid, the authors demonstrated the differences of isomeric sialyl-*N*-glycans between healthy and diseased groups. In comparison to healthy donors, BD patients possessed more α2,3-linked sialic acid on their *N*-glycans, a novel, potential molecular signature for the diagnosis of BD.

Mass spectrometry has become the most useful tool for studying glycomes. Different analytical approaches have been established to describe, in addition to glycan profiles based on monosaccharide composition, also linkage and anomeric information. Previously, Dr. Kay-Hooi Khoo and collaborators have demonstrated that precise glycomic profiles of different zebrafish organs can be mapped using mass spectrometry (Yamakawa et al., 2018). As a complementary study, Tseng et al. employed a nano-LC-MS/MS-based analytical workflow to re-examine zebrafish *N*- and *O*-glycans that have undergone permethylation. This approach is highly sensitive due to an increased ionization efficiency of glycans after derivatization and is advantageous for identifying isomeric structures using diagnostic fragmentation ions. Interestingly, a repertoire of glycans with sulfate substitution was revealed in the negative ion mode, including sulfated Lac(di-)NAc and sialyl-LacNAc epitopes on *N*-glycans and sulfated core 1 *O*-glycan structures. Using a very similar analytical approach, Aoki et al. compared the serum *N*-glycomes of five fish species (teleost and chondrostrean). Despite the genomes remaining unsequenced, the analysis showed these fishes share basic *N*-glycan biosynthetic pathways with other vertebrates, as indicated by the presence of high-mannose type and complex-type *N*-glycans. The authors discovered novel motifs on the *N*-glycan antennae, featuring different sialylation patterns, degrees of *O*-acetylation of sialic acid and galactose modifications. To provide linkage details of the terminal residues, the authors also employed exo-glycosidases to digest glycopeptides prior to glycan analysis. This led to the discovery of a species-specific α-galactose modification, which was only found in the two sturgeon spices.

In contrast to sialic acid-bearing glycans that are most frequently observed in vertebrates and some pathogens, the deoxyhexose sugar, fucose, has been found in a wider variety of organisms. Thomès and Bojar looked into the distribution of fucose-containing glycans in over 2,000 organisms covering all taxonomic kingdoms. Using a machine learning approach, the authors thoroughly analyzed glycan structures stored in public databases and described in peer-reviewed literature. Interestingly, bacterial and fungal species were able to make glycans with the highest number of fucose residues, whereas fucose was often attached to *N*-acetyl-glucosamine in all kingdoms except fungi. The authors also explored the correlation of fucose usage in different *E. coli* strains and their growing environment as well as infection potential.

## Biosynthesis of α-Gal Epitope in Mammals and Ticks

The galactose-α-1,3-galactose (α-Gal) is a carbohydrate antigen abundantly expressed on glycoproteins and glycolipids in cells of non-primate mammals, prosimians, and New World monkeys. However, humans and crown catarrhines evolved with the inability to synthesize the glycan molecule due to the functional inactivation of the α1,3-galactosyltransferase (α1,3GalT). In turn, they can naturally produce antibodies specific to α-Gal that may be harnessed for the development of novel immunotherapies. In his comprehensive review article, Galili described the immunological processes associated with anti-Gal/α-Gal epitope interactions and suggested α-Gal therapies based on these immune reactions, which include *i*) amplification of whole virus vaccine immunogenicity by α-Gal epitopes; *ii*) conversion of autologous tumors into antitumor vaccines by expression of α-gal epitopes on tumor cell membranes; *iii*) accelerating healing of external and internal injuries by α-Gal nanoparticles which decrease the healing time and diminish scar formation; and *iv*) increasing anti-Gal–mediated protection against pathogens carrying α-Gal or α-Gal-like epitopes.

Despite the potential beneficial effects of the α-Gal immunity, exposure to the α-Gal epitope from the saliva of certain tick species induces a complex allergic disease (i.e. α-Gal syndrome or red meat allergy) characterized by the development of specific anti-α-Gal IgE antibodies after mammalian meat consumption. However, the origin of the glyco-epitope in ticks is still a matter of scientific debate. The work by Sharma et al. investigated the functional role of the *α*-D-galactosidase (ADGal) and β-1,4 galactosyltransferase (β-1,4GalT) in endogenous α-Gal production, carbohydrate metabolism, and *N*-glycans profile in the *Amblyomma americanum* tick. Their study provides an insight into tick intrinsic factors involved in synthesizing or recycling of α-Gal and its possible involvement in the onset of the α-Gal syndrome.

## Future Aspect

We hope that the articles in this series will provide the glyco-crowd with examples of the power of high-resolution mass spectrometry in characterizing novel glyco-epitopes and will improve the understanding of the structural diversity of glycans in different organisms, while bringing more insights into the biosynthesis and biological roles of the fascinating sugar molecules.
